# {3,3′,5,5′-Tetra­meth­oxy-2,2′-[ethane-1,2-diylbis(nitrilo­methyl­idyne)]diphenolato}nickel(II)

**DOI:** 10.1107/S1600536810016041

**Published:** 2010-05-08

**Authors:** Gervas E. Assey, Ray J. Butcher, Yilma Gultneh

**Affiliations:** a Department of Chemistry, Howard University, 525 College Street NW, Washington, DC 20059, USA

## Abstract

The title square-planar nickel complex, [Ni(C_20_H_22_N_2_O_6_)], has Ni—N and Ni—O bond lengths of 1.8448 (14)/1.8478 (14) and 1.8536 (12)/1.8520 (12) Å. There is a slight twist in the two benzene rings at each end of the complex [dihedral angle = 11.11 (5)°]. All the atoms of the meth­oxy substitutents are in the plane of the ring to which they are attached except for one which deviates slightly [0.365 (3) Å]. In the crystal, weak C—H⋯O inter­molecular inter­actions connect the mol­ecules.

## Related literature

For nickel–salen complexes with aromatic substituents, see: Bal & Ülküseven (2004 ▶). For their activation of O_2_, see: Soto-Garrodo & Salas-Reyes (2000 ▶); For their catalytic activity: Silva *et al.* (2002 ▶); Santos *et al.* (2000 ▶); Yoon & Burrows (1988 ▶). For the mesogenic properties of substituted complexes, see: Blake *et al.* (1995 ▶). For bond-length data, see: Allen *et al.* (1987 ▶). 
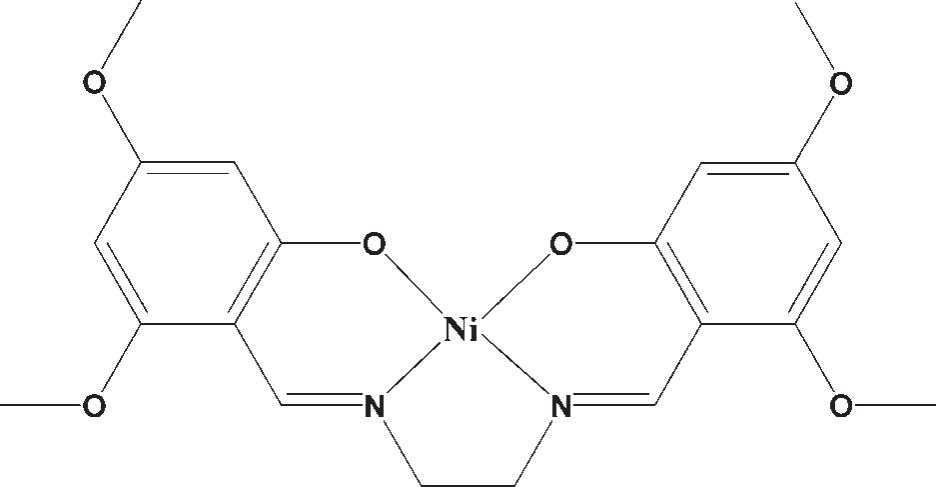


## Experimental

### 

#### Crystal data

[Ni(C_20_H_22_N_2_O_6_)]
*M* *_r_* = 445.11
Monoclinic, 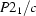

*a* = 7.41599 (12) Å
*b* = 15.6945 (2) Å
*c* = 15.7203 (2) Å
β = 91.9153 (13)°
*V* = 1828.67 (5) Å^3^
*Z* = 4
Cu *K*α radiation
μ = 1.91 mm^−1^
*T* = 110 K
0.53 × 0.15 × 0.12 mm

#### Data collection

Oxford Diffraction Xcalibur Ruby Gemini diffractometer
Absorption correction: multi-scan (*CrysAlis PRO*; Oxford Diffraction, 2007 ▶) *T*_min_ = 0.602, *T*_max_ = 1.000
6746 measured reflections
3603 independent reflections
3370 reflections with *I* > 2σ(*I*)
*R*_int_ = 0.022

#### Refinement

*R*[*F* ^2^ > 2σ(*F* ^2^)] = 0.036
*wR*(*F* ^2^) = 0.098
*S* = 1.04
3603 reflections
266 parameters
H-atom parameters constrained
Δρ_max_ = 0.42 e Å^−3^
Δρ_min_ = −0.47 e Å^−3^

### 

Data collection: *CrysAlis PRO* (Oxford Diffraction, 2007 ▶); cell refinement: *CrysAlis PRO*; data reduction: *CrysAlis PRO*; program(s) used to solve structure: *SHELXS97* (Sheldrick, 2008 ▶); program(s) used to refine structure: *SHELXL97* (Sheldrick, 2008 ▶); molecular graphics: *SHELXTL* (Sheldrick, 2008 ▶); software used to prepare material for publication: *SHELXTL*.

## Supplementary Material

Crystal structure: contains datablocks I, global. DOI: 10.1107/S1600536810016041/bt5260sup1.cif

Structure factors: contains datablocks I. DOI: 10.1107/S1600536810016041/bt5260Isup2.hkl

Additional supplementary materials:  crystallographic information; 3D view; checkCIF report

## Figures and Tables

**Table 1:** Hydrogen-bond geometry (Å, °)

*D*—H⋯*A*	*D*—H	H⋯*A*	*D*⋯*A*	*D*—H⋯*A*
C8—H8*B*⋯O2^i^	0.99	2.61	3.587 (2)	169
C17—H17*A*⋯O4^ii^	0.98	2.60	3.484 (2)	150

Symmetry codes: (i) 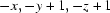; (ii) 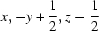.

**Fig. 1.**
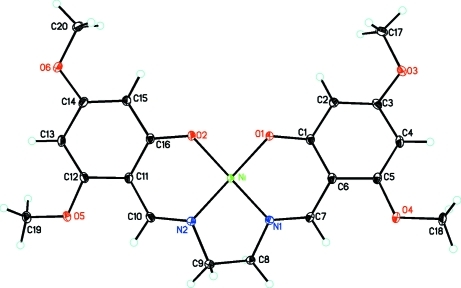
Diagram of the square planar nickel complex C20H22N2NiO6 showing atom labeling.

**Fig. 2.**
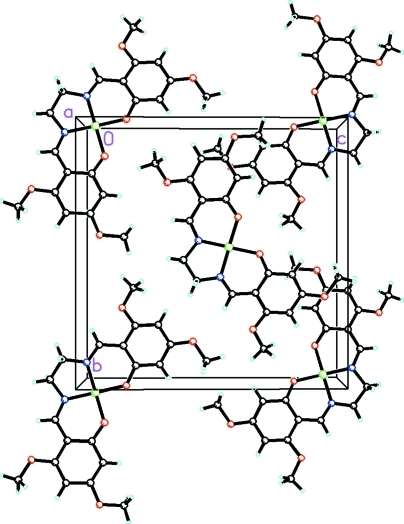
The molecular packing for C20H22N2NiO6 viewed down the a axis.

## supplementary crystallographic information

### Comment

Complexes of Ni(II) with N_2_O_2_ Schiff bases derived from salicylaldehyde have been studied for a long time as homogeneous catalysts due to their high activity and selectivity (Silva *et al.* 2002, Santos *et al.* 2000). These types of complexes have been described in the literature as catalytically active in oxidation and reduction reactions both as homogeneous and heterogeneous catalysts (Yoon & Burrows, 1988). Other areas where coordination chemistry has found application are in the areas of molecular adsorption, liquid-liquid extraction, ion exchange and metalloenzymes. They have been designed as potential transition metal host systems in host-guest chemistry in the similar way to that of enzyme substrate.

The importance of nickel salen complexes with aromatic substituents range from biological (Bal and Ülküseven, 2004), activation of O_2_ under very mild conditions (Soto-Garrodo, & Salas-Reyes, 2000) and mesogenic properties of substituted complexes. (Blake *et al.* 1995) reported metallomesogens based on nickel alkyl and alkoxy substituted salen.

The central Ni is in a square planar coordination environment of O1 O2, N1 and N2 with rms deviation of 0.0461 (6) Å (deviation from plane for Ni of 0.0034 (7) Å). The Ni—N and Ni—O bond distances are in the normal range for Ni-salen type complexes (Allen *et al.*, 1987) at 1.8448 (14), and 1.8478 (14) Å for Ni—N and 1.8536 (12) Å and 1.8520 (12) Å for Ni—O. There is a slight twist in the two phenyl rings at each end of the complex (dihedral angle of 11.11 (5)°. All the atoms of the methoxy substitutents are in the plane of the ring to which they are attached except C19 which deviates slightly (0.365 (3) °). There are weak C—H···O intermolecular interactions connecting  the molecules in the solid state.

### Experimental

The ligand synthesis was accomplished by adding a solution of (2 g, 33.3 mmol) ethylenediamine in 25 mls of methanol to a solution of (12.13 g, 66.6 mmol) 4,6-dimethoxysalicylaldehyde in 40 ml of methanol. The mixture was refluxed overnight while stirring. Then the mixture was evaporated under reduced pressure to afford yellow solids.

The complex was synthesized by mixing a solution of (0.38 g, 1 mmol) *N*,*N*-ethylenebis(4,6-dimethoxysalicylaldimine) in 5 ml of CH_2_Cl_2_ with a solution of (0.29 g, 1 mmol) nickel nitrate hexahydrate in 5 ml methanol. The solution mixture was stirred for 1 hour then filtered and layered with diethyl ether for crystallization. Single crystals of X-ray quality were obtained.

### Refinement

H atoms were placed in geometrically idealized positions and constrained to ride on their parent atoms with a C—H distances of 0.95 and 0.99 Å *U*_iso_(H) = 1.2*U*_eq_(C) and 0.98 Å for CH_3_ [*U*_iso_(H) = 1.5*U*_eq_(C)].

### Crystal data

**Table:**

[Ni(C_20_H_22_N_2_O_6_)]	*F*(000) = 928
*M**_r_* = 445.11	*D*_x_ = 1.617 Mg m^−^^3^
Monoclinic, *P*2_1_/*c*	Cu *K*α radiation, λ = 1.54184 Å
Hall symbol: -P 2ybc	Cell parameters from 5663 reflections
*a* = 7.41599 (12) Å	θ = 5.6–74.0°
*b* = 15.6945 (2) Å	µ = 1.91 mm^−^^1^
*c* = 15.7203 (2) Å	*T* = 110 K
β = 91.9153 (13)°	Needle, red
*V* = 1828.67 (5) Å^3^	0.53 × 0.15 × 0.12 mm
*Z* = 4	

### Data collection

**Table:**

Oxford Diffraction Xcalibur Ruby Gemini diffractometer	3603 independent reflections
Radiation source: Enhance (Cu) X-ray Source	3370 reflections with *I* > 2σ(*I*)
graphite	*R*_int_ = 0.022
Detector resolution: 10.5081 pixels mm^-1^	θ_max_ = 74.2°, θ_min_ = 5.6°
ω scans	*h* = −9→8
Absorption correction: multi-scan (*CrysAlis PRO*; Oxford Diffraction, 2007)	*k* = −11→19
*T*_min_ = 0.602, *T*_max_ = 1.000	*l* = −18→19
6746 measured reflections	

### Refinement

**Table:**

Refinement on *F*^2^	Primary atom site location: structure-invariant direct methods
Least-squares matrix: full	Secondary atom site location: difference Fourier map
*R*[*F*^2^ > 2σ(*F*^2^)] = 0.036	Hydrogen site location: inferred from neighbouring sites
*wR*(*F*^2^) = 0.098	H-atom parameters constrained
*S* = 1.04	*w* = 1/[σ^2^(*F*_o_^2^) + (0.0641*P*)^2^ + 1.0294*P*] where *P* = (*F*_o_^2^ + 2*F*_c_^2^)/3
3603 reflections	(Δ/σ)_max_ < 0.001
266 parameters	Δρ_max_ = 0.42 e Å^−^^3^
0 restraints	Δρ_min_ = −0.47 e Å^−^^3^

### Special details

**Table:**

Geometry. All esds (except the esd in the dihedral angle between two l.s. planes) are estimated using the full covariance matrix. The cell esds are taken into account individually in the estimation of esds in distances, angles and torsion angles; correlations between esds in cell parameters are only used when they are defined by crystal symmetry. An approximate (isotropic) treatment of cell esds is used for estimating esds involving l.s. planes.
Refinement. Refinement of *F*^2^ against ALL reflections. The weighted *R*-factor wR and goodness of fit *S* are based on *F*^2^, conventional *R*-factors *R* are based on *F*, with *F* set to zero for negative *F*^2^. The threshold expression of *F*^2^ > σ(*F*^2^) is used only for calculating *R*-factors(gt) etc. and is not relevant to the choice of reflections for refinement. *R*-factors based on *F*^2^ are statistically about twice as large as those based on *F*, and *R*- factors based on ALL data will be even larger.

### Fractional atomic coordinates and isotropic or equivalent isotropic displacement parameters (Å^2^)

**Table:**

	*x*	*y*	*z*	*U*_iso_*/*U*_eq_	
Ni	0.25525 (4)	0.522587 (16)	0.436449 (16)	0.01249 (11)	
O1	0.25637 (17)	0.49637 (8)	0.32150 (7)	0.0166 (2)	
O2	0.25493 (17)	0.63580 (7)	0.40321 (7)	0.0168 (2)	
O3	0.36236 (18)	0.33461 (8)	0.07279 (8)	0.0209 (3)	
O4	0.45100 (16)	0.20821 (7)	0.34353 (7)	0.0183 (3)	
O5	0.10218 (18)	0.76296 (8)	0.66590 (7)	0.0192 (3)	
O6	0.13141 (19)	0.93215 (8)	0.41651 (8)	0.0230 (3)	
N1	0.26710 (19)	0.41000 (9)	0.46989 (8)	0.0143 (3)	
N2	0.24084 (18)	0.54755 (10)	0.55089 (9)	0.0145 (3)	
C1	0.3028 (2)	0.42368 (10)	0.28756 (10)	0.0142 (3)	
C2	0.3067 (2)	0.41965 (10)	0.19756 (10)	0.0158 (3)	
H2A	0.2743	0.4682	0.1643	0.019*	
C3	0.3576 (2)	0.34513 (11)	0.15833 (10)	0.0163 (3)	
C4	0.4124 (2)	0.27279 (11)	0.20515 (11)	0.0171 (3)	
H4A	0.4539	0.2233	0.1771	0.020*	
C5	0.4047 (2)	0.27513 (10)	0.29228 (11)	0.0150 (3)	
C6	0.3462 (2)	0.34938 (10)	0.33579 (11)	0.0152 (3)	
C7	0.3154 (2)	0.34535 (10)	0.42475 (11)	0.0145 (3)	
H7A	0.3313	0.2920	0.4526	0.017*	
C8	0.2141 (2)	0.39623 (11)	0.55814 (10)	0.0164 (3)	
H8A	0.2732	0.3445	0.5820	0.020*	
H8B	0.0818	0.3891	0.5605	0.020*	
C9	0.2745 (2)	0.47426 (10)	0.60768 (11)	0.0171 (3)	
H9A	0.2051	0.4801	0.6601	0.020*	
H9B	0.4044	0.4702	0.6239	0.020*	
C10	0.2071 (2)	0.62123 (11)	0.58443 (10)	0.0155 (3)	
H10A	0.1973	0.6239	0.6445	0.019*	
C11	0.1837 (2)	0.69804 (11)	0.53782 (10)	0.0155 (3)	
C12	0.1328 (2)	0.77404 (11)	0.58121 (10)	0.0165 (3)	
C13	0.1162 (2)	0.85044 (11)	0.53987 (11)	0.0188 (3)	
H13A	0.0831	0.9005	0.5695	0.023*	
C14	0.1498 (2)	0.85285 (11)	0.45184 (11)	0.0171 (3)	
C15	0.1950 (2)	0.78148 (11)	0.40677 (10)	0.0165 (3)	
H15A	0.2138	0.7853	0.3474	0.020*	
C16	0.2134 (2)	0.70230 (10)	0.44895 (10)	0.0144 (3)	
C17	0.3271 (3)	0.40816 (12)	0.02106 (11)	0.0264 (4)	
H17A	0.3333	0.3925	−0.0391	0.040*	
H17B	0.4174	0.4521	0.0347	0.040*	
H17C	0.2065	0.4302	0.0322	0.040*	
C18	0.5069 (3)	0.13184 (11)	0.30218 (12)	0.0213 (4)	
H18A	0.5485	0.0903	0.3451	0.032*	
H18B	0.6056	0.1450	0.2643	0.032*	
H18C	0.4050	0.1080	0.2688	0.032*	
C19	0.0109 (2)	0.83006 (11)	0.70858 (11)	0.0203 (4)	
H19A	−0.0207	0.8109	0.7655	0.030*	
H19B	−0.0992	0.8453	0.6759	0.030*	
H19C	0.0901	0.8799	0.7136	0.030*	
C20	0.1620 (3)	0.93901 (11)	0.32755 (12)	0.0232 (4)	
H20A	0.1452	0.9983	0.3094	0.035*	
H20B	0.0763	0.9025	0.2958	0.035*	
H20C	0.2855	0.9209	0.3164	0.035*	

### Atomic displacement parameters (Å^2^)

**Table:**

	*U*^11^	*U*^22^	*U*^33^	*U*^12^	*U*^13^	*U*^23^
Ni	0.01826 (18)	0.00876 (17)	0.01067 (17)	0.00064 (10)	0.00377 (11)	−0.00065 (9)
O1	0.0266 (6)	0.0109 (5)	0.0127 (5)	0.0024 (5)	0.0038 (4)	−0.0009 (4)
O2	0.0253 (6)	0.0111 (5)	0.0144 (6)	0.0006 (5)	0.0071 (4)	−0.0013 (4)
O3	0.0311 (7)	0.0181 (6)	0.0139 (6)	0.0004 (5)	0.0046 (5)	−0.0034 (5)
O4	0.0253 (6)	0.0112 (5)	0.0185 (6)	0.0045 (5)	0.0019 (5)	−0.0026 (4)
O5	0.0298 (7)	0.0148 (6)	0.0135 (6)	0.0034 (5)	0.0054 (5)	−0.0033 (4)
O6	0.0381 (8)	0.0110 (6)	0.0205 (6)	0.0021 (5)	0.0089 (5)	0.0003 (5)
N1	0.0170 (7)	0.0130 (6)	0.0130 (6)	0.0002 (5)	0.0033 (5)	0.0006 (5)
N2	0.0174 (7)	0.0133 (7)	0.0129 (6)	0.0001 (5)	0.0024 (5)	0.0007 (5)
C1	0.0143 (7)	0.0117 (7)	0.0167 (8)	−0.0022 (6)	0.0027 (6)	−0.0022 (6)
C2	0.0188 (8)	0.0128 (8)	0.0158 (8)	−0.0016 (6)	0.0029 (6)	−0.0002 (6)
C3	0.0162 (8)	0.0180 (8)	0.0149 (8)	−0.0035 (6)	0.0045 (6)	−0.0033 (6)
C4	0.0178 (8)	0.0137 (8)	0.0200 (8)	−0.0006 (6)	0.0042 (6)	−0.0061 (6)
C5	0.0138 (7)	0.0118 (7)	0.0195 (8)	−0.0010 (6)	0.0020 (6)	−0.0022 (6)
C6	0.0156 (7)	0.0127 (8)	0.0175 (8)	−0.0012 (6)	0.0023 (6)	−0.0025 (6)
C7	0.0154 (8)	0.0103 (7)	0.0178 (8)	0.0005 (6)	0.0012 (6)	−0.0004 (6)
C8	0.0229 (8)	0.0131 (8)	0.0134 (8)	0.0023 (6)	0.0046 (6)	0.0025 (6)
C9	0.0229 (9)	0.0161 (8)	0.0122 (7)	0.0024 (6)	0.0023 (6)	0.0011 (6)
C10	0.0176 (8)	0.0164 (8)	0.0128 (7)	−0.0002 (6)	0.0031 (6)	−0.0025 (6)
C11	0.0173 (8)	0.0127 (8)	0.0166 (8)	−0.0009 (6)	0.0022 (6)	−0.0030 (6)
C12	0.0192 (8)	0.0159 (8)	0.0145 (7)	−0.0010 (6)	0.0028 (6)	−0.0039 (6)
C13	0.0244 (9)	0.0127 (8)	0.0196 (8)	0.0001 (6)	0.0044 (6)	−0.0051 (6)
C14	0.0202 (8)	0.0109 (7)	0.0204 (8)	−0.0006 (6)	0.0027 (6)	0.0006 (6)
C15	0.0213 (8)	0.0131 (8)	0.0154 (7)	−0.0019 (6)	0.0050 (6)	−0.0012 (6)
C16	0.0148 (7)	0.0118 (7)	0.0169 (8)	−0.0026 (6)	0.0037 (6)	−0.0023 (6)
C17	0.0445 (12)	0.0206 (9)	0.0144 (8)	−0.0075 (8)	0.0067 (7)	0.0003 (7)
C18	0.0266 (9)	0.0127 (8)	0.0249 (9)	0.0053 (7)	0.0051 (7)	−0.0042 (6)
C19	0.0239 (9)	0.0187 (8)	0.0186 (8)	0.0024 (7)	0.0061 (6)	−0.0051 (6)
C20	0.0318 (10)	0.0153 (8)	0.0231 (9)	0.0041 (7)	0.0105 (7)	0.0053 (7)

### Geometric parameters (Å, °)

**Table:**

Ni—N1	1.8448 (14)	C8—C9	1.511 (2)
Ni—N2	1.8478 (14)	C8—H8A	0.9900
Ni—O2	1.8520 (12)	C8—H8B	0.9900
Ni—O1	1.8536 (12)	C9—H9A	0.9900
O1—C1	1.310 (2)	C9—H9B	0.9900
O2—C16	1.310 (2)	C10—C11	1.418 (2)
O3—C3	1.356 (2)	C10—H10A	0.9500
O3—C17	1.431 (2)	C11—C16	1.423 (2)
O4—C5	1.361 (2)	C11—C12	1.431 (2)
O4—C18	1.4316 (19)	C12—C13	1.368 (2)
O5—C12	1.369 (2)	C13—C14	1.415 (2)
O5—C19	1.4311 (19)	C13—H13A	0.9500
O6—C14	1.368 (2)	C14—C15	1.373 (2)
O6—C20	1.428 (2)	C15—C16	1.413 (2)
N1—C7	1.295 (2)	C15—H15A	0.9500
N1—C8	1.4705 (19)	C17—H17A	0.9800
N2—C10	1.299 (2)	C17—H17B	0.9800
N2—C9	1.472 (2)	C17—H17C	0.9800
C1—C2	1.418 (2)	C18—H18A	0.9800
C1—C6	1.422 (2)	C18—H18B	0.9800
C2—C3	1.381 (2)	C18—H18C	0.9800
C2—H2A	0.9500	C19—H19A	0.9800
C3—C4	1.406 (2)	C19—H19B	0.9800
C4—C5	1.373 (2)	C19—H19C	0.9800
C4—H4A	0.9500	C20—H20A	0.9800
C5—C6	1.426 (2)	C20—H20B	0.9800
C6—C7	1.426 (2)	C20—H20C	0.9800
C7—H7A	0.9500		
			
N1—Ni—N2	85.95 (6)	N2—C9—H9B	110.5
N1—Ni—O2	177.34 (6)	C8—C9—H9B	110.5
N2—Ni—O2	94.11 (6)	H9A—C9—H9B	108.7
N1—Ni—O1	93.64 (6)	N2—C10—C11	124.68 (15)
N2—Ni—O1	176.89 (6)	N2—C10—H10A	117.7
O2—Ni—O1	86.44 (5)	C11—C10—H10A	117.7
C1—O1—Ni	126.87 (11)	C10—C11—C16	121.80 (15)
C16—O2—Ni	127.42 (10)	C10—C11—C12	119.46 (15)
C3—O3—C17	117.05 (13)	C16—C11—C12	118.73 (15)
C5—O4—C18	116.65 (13)	C13—C12—O5	123.95 (15)
C12—O5—C19	117.32 (13)	C13—C12—C11	121.68 (15)
C14—O6—C20	116.69 (13)	O5—C12—C11	114.37 (14)
C7—N1—C8	119.28 (14)	C12—C13—C14	118.19 (15)
C7—N1—Ni	127.27 (12)	C12—C13—H13A	120.9
C8—N1—Ni	113.45 (10)	C14—C13—H13A	120.9
C10—N2—C9	118.73 (13)	O6—C14—C15	123.75 (15)
C10—N2—Ni	127.03 (12)	O6—C14—C13	113.77 (15)
C9—N2—Ni	114.24 (11)	C15—C14—C13	122.48 (15)
O1—C1—C2	117.36 (14)	C14—C15—C16	119.78 (15)
O1—C1—C6	123.69 (15)	C14—C15—H15A	120.1
C2—C1—C6	118.94 (14)	C16—C15—H15A	120.1
C3—C2—C1	119.91 (15)	O2—C16—C15	117.64 (14)
C3—C2—H2A	120.0	O2—C16—C11	123.25 (15)
C1—C2—H2A	120.0	C15—C16—C11	119.11 (15)
O3—C3—C2	124.24 (16)	O3—C17—H17A	109.5
O3—C3—C4	113.82 (15)	O3—C17—H17B	109.5
C2—C3—C4	121.94 (15)	H17A—C17—H17B	109.5
C5—C4—C3	118.66 (15)	O3—C17—H17C	109.5
C5—C4—H4A	120.7	H17A—C17—H17C	109.5
C3—C4—H4A	120.7	H17B—C17—H17C	109.5
O4—C5—C4	123.50 (15)	O4—C18—H18A	109.5
O4—C5—C6	114.92 (14)	O4—C18—H18B	109.5
C4—C5—C6	121.58 (15)	H18A—C18—H18B	109.5
C1—C6—C7	121.24 (15)	O4—C18—H18C	109.5
C1—C6—C5	118.77 (15)	H18A—C18—H18C	109.5
C7—C6—C5	119.70 (15)	H18B—C18—H18C	109.5
N1—C7—C6	123.96 (15)	O5—C19—H19A	109.5
N1—C7—H7A	118.0	O5—C19—H19B	109.5
C6—C7—H7A	118.0	H19A—C19—H19B	109.5
N1—C8—C9	106.45 (13)	O5—C19—H19C	109.5
N1—C8—H8A	110.4	H19A—C19—H19C	109.5
C9—C8—H8A	110.4	H19B—C19—H19C	109.5
N1—C8—H8B	110.4	O6—C20—H20A	109.5
C9—C8—H8B	110.4	O6—C20—H20B	109.5
H8A—C8—H8B	108.6	H20A—C20—H20B	109.5
N2—C9—C8	106.22 (13)	O6—C20—H20C	109.5
N2—C9—H9A	110.5	H20A—C20—H20C	109.5
C8—C9—H9A	110.5	H20B—C20—H20C	109.5
			
N1—Ni—O1—C1	−16.22 (14)	O4—C5—C6—C7	−8.9 (2)
N2—Ni—O1—C1	−98.5 (11)	C4—C5—C6—C7	171.01 (15)
O2—Ni—O1—C1	161.12 (14)	C8—N1—C7—C6	171.34 (15)
N1—Ni—O2—C16	−105.1 (12)	Ni—N1—C7—C6	−8.6 (2)
N2—Ni—O2—C16	−13.82 (14)	C1—C6—C7—N1	−8.2 (3)
O1—Ni—O2—C16	163.11 (14)	C5—C6—C7—N1	177.99 (16)
N2—Ni—N1—C7	−165.37 (15)	C7—N1—C8—C9	146.29 (15)
O2—Ni—N1—C7	−73.9 (12)	Ni—N1—C8—C9	−33.74 (16)
O1—Ni—N1—C7	17.72 (15)	C10—N2—C9—C8	151.31 (15)
N2—Ni—N1—C8	14.66 (12)	Ni—N2—C9—C8	−29.43 (16)
O2—Ni—N1—C8	106.1 (12)	N1—C8—C9—N2	38.49 (17)
O1—Ni—N1—C8	−162.25 (11)	C9—N2—C10—C11	175.76 (15)
N1—Ni—N2—C10	−171.54 (15)	Ni—N2—C10—C11	−3.4 (3)
O2—Ni—N2—C10	11.12 (15)	N2—C10—C11—C16	−6.4 (3)
O1—Ni—N2—C10	−89.0 (11)	N2—C10—C11—C12	175.06 (16)
N1—Ni—N2—C9	9.28 (12)	C19—O5—C12—C13	14.1 (2)
O2—Ni—N2—C9	−168.06 (11)	C19—O5—C12—C11	−165.63 (15)
O1—Ni—N2—C9	91.8 (11)	C10—C11—C12—C13	177.11 (16)
Ni—O1—C1—C2	−175.30 (11)	C16—C11—C12—C13	−1.5 (3)
Ni—O1—C1—C6	5.7 (2)	C10—C11—C12—O5	−3.2 (2)
O1—C1—C2—C3	178.92 (15)	C16—C11—C12—O5	178.18 (14)
C6—C1—C2—C3	−2.0 (2)	O5—C12—C13—C14	−179.25 (16)
C17—O3—C3—C2	5.6 (2)	C11—C12—C13—C14	0.4 (3)
C17—O3—C3—C4	−173.47 (16)	C20—O6—C14—C15	−0.1 (3)
C1—C2—C3—O3	178.78 (15)	C20—O6—C14—C13	−179.51 (15)
C1—C2—C3—C4	−2.2 (3)	C12—C13—C14—O6	−179.43 (16)
O3—C3—C4—C5	−177.02 (15)	C12—C13—C14—C15	1.1 (3)
C2—C3—C4—C5	3.9 (2)	O6—C14—C15—C16	179.13 (16)
C18—O4—C5—C4	−1.1 (2)	C13—C14—C15—C16	−1.5 (3)
C18—O4—C5—C6	178.73 (14)	Ni—O2—C16—C15	−170.27 (11)
C3—C4—C5—O4	178.62 (15)	Ni—O2—C16—C11	8.7 (2)
C3—C4—C5—C6	−1.2 (2)	C14—C15—C16—O2	179.34 (15)
O1—C1—C6—C7	9.7 (3)	C14—C15—C16—C11	0.3 (2)
C2—C1—C6—C7	−169.32 (15)	C10—C11—C16—O2	3.6 (3)
O1—C1—C6—C5	−176.52 (15)	C12—C11—C16—O2	−177.85 (15)
C2—C1—C6—C5	4.5 (2)	C10—C11—C16—C15	−177.45 (15)
O4—C5—C6—C1	177.22 (14)	C12—C11—C16—C15	1.1 (2)
C4—C5—C6—C1	−2.9 (2)		

### Hydrogen-bond geometry (Å, °)

**Table:**

*D*—H···*A*	*D*—H	H···*A*	*D*···*A*	*D*—H···*A*
C8—H8B···O2^i^	0.99	2.61	3.587 (2)	169
C17—H17A···O4^ii^	0.98	2.60	3.484 (2)	150

Symmetry codes: (i) −*x*, −*y*+1, −*z*+1; (ii) *x*, −*y*+1/2, *z*−1/2.
